# Persistence, Dosing, and Other Treatment Patterns Among Crohn’s Disease Patients Initiating Biologics in United States

**DOI:** 10.1093/crocol/otab076

**Published:** 2021-11-05

**Authors:** Amanda Teeple, Janvi Sah, Rajesh Mallampati, Christopher Adams, Dexter Waters, Erik Muser

**Affiliations:** 1 Janssen Scientific Affairs, Real World Value & Evidence, Horsham, Pennsylvania, USA; 2 STATinMED Research, Health Economics and Outcomes Research, Ann Arbor, Michigan, USA

**Keywords:** Crohn’s disease, ustekinumab, anti-TNF biologics, persistence, dose titration

## Abstract

**Background:**

This study examined biologic persistence, dosing, and other treatment patterns among Crohn’s disease (CD) patients that initiated adalimumab (ADA), certolizumab pegol (CZP), infliximab (IFX), ustekinumab (UST), and vedolizumab (VDZ).

**Methods:**

This descriptive study pooled data from IBM MarketScan, IQVIA PharMetrics, and Optum databases and identified CD patients who initiated the above biologics. Due to low sample size, CZP was not included in the analyses. Persistence was defined as the proportion of patients that remained on the index biologic without a gap of >30 days for ADA and >120 days for UST, IFX, and VDZ between two claims. A sensitivity analysis using fixed gap (90-day) was also conducted. Dose titration (based upon mean maintenance dose) including 50% dose escalation, and 50% dose reduction was assessed among patients with ≥2 maintenance doses during follow-up among ADA, UST, and VDZ patients.

**Results:**

After applying all selection criteria, patients were selected into bio-naive (ADA: 2047; IFX: 1127; UST: 296; VDZ: 342) and bio-experienced cohorts (ADA: 300; IFX: 341; UST: 801; VDZ: 593) based on the biologics used. Unadjusted persistence was numerically higher among bio-naive and bio-experienced UST (87.2%, 86.3%) patients followed by VDZ (78.9%, 80.8%), IFX (79.0%, 77.4%), and ADA (64.9%, 60.7%). Similar trends were observed using sensitivity analysis. Dose escalation was numerically higher for ADA patients (16.1%–16.4%) followed by UST (13.4%–16.9%), and VDZ (12.4%–14.7%). Dose reduction followed a similar trend.

**Conclusions:**

Among CD patients, unadjusted persistence using variable and fixed gap definition was numerically highest for UST patients whereas dose escalation was numerically highest among ADA patients. Further research is needed to examine treatment patterns after adjusting for confounders and baseline differences among biologic users.

## Introduction

Crohn’s disease (CD) is a chronic disease characterized by transmural inflammation and can involve any portion of the gastrointestinal tract. The disease presentation can vary and includes abdominal pain, fever, weight loss, and clinical bowel obstruction.^1^ The annual incidence of CD in the United States is ~20 cases per 100000 persons.^2,3^ Adequate treatment is crucial, as CD is incurable and can relapse over time which may lead to bowel damage. Clinical management aims to interrupt the destructive progression of CD to achieve remission.^1,4^

Antitumor necrosis factor (TNF) agents (adalimumab [ADA], certolizumab pegol [CZP], infliximab [IFX]), vedolizumab [VDZ], and ustekinumab [UST]) which target specific immunological pathways have demonstrated effectiveness in relieving disease burden in CD patients, particularly for those with an inadequate response or intolerance to prior conventional therapies such as oral corticosteroids and immunomodulators.^3–6^ Variations in toleration and response to treatment have been noted with biologics, resulting in dose adjustments, augmentation, therapy changes, and discontinuation.^7^ A real-world study in CD patients reported a persistence rate of 51% for ADA, 48% for IFX, and 35% for CZP over 12 months with VDZ results not reported due to the small sample size.^8^ A recent systematic literature review found that dose escalation was as high as 29.9% among CD patients that initiated treatment with ADA and IFX.^9^ The escalation rates were different for bio-naive and bio-experienced patients since the rates increased according to line of therapy—19% for first line, 37% for second line, and 41% for third line of treatment.^9^

The most recent 2018 American College of Gastroenterology guidelines recommend the use of UST in patients who failed previous treatment with corticosteroids, thiopurines, methotrexate, or anti-TNF inhibitors or who have had no prior exposure to anti-TNF inhibitors.^10^ Persistence and treatment patterns for biologics can serve as useful proxies for their effectiveness in CD patients, and real-world evidence on such endpoints remains limited for UST in CD. This study aimed to descriptively evaluate biologic persistence, dose titration, and other treatment patterns in bio-naive and bio-experienced CD patients using three large commercial health care insurance claims databases.

## Methods

### Data Source

This study pooled data from three US commercial databases: IBM MarketScan (IBM; September 26, 2015–September 30, 2018), the Optum Clinformatics Data Mart (Optum: September 26, 2015–March 31, 2019), and IQVIA PharMetrics Plus (IQVIA; September 26, 2015–December 31, 2018). These databases contain medical and pharmacy claims for commercial populations in the United States. The medical claims were coded using International Classification of Disease, 9th Revision, Clinical Modification (ICD-9-CM), ICD-10-CM (implemented on October 1, 2015), or Current Procedural Terminology (CPT) whereas the pharmacy claims were coded using National Drug Code (NDC) or Health Care Common Procedure Coding Systems (HCPCS). Among all the biologics approved for the treatment of moderately-to-severely active CD, UST was the most recent biologic approved by the US Food and Drug Administration (FDA) on September 23, 2016. Hence, the following Monday (September 26, 2016) was used as the start of the patient identification period. All databases used were independent and contained no personal identifiable information.

### Patient Selection

**Figure 1.**
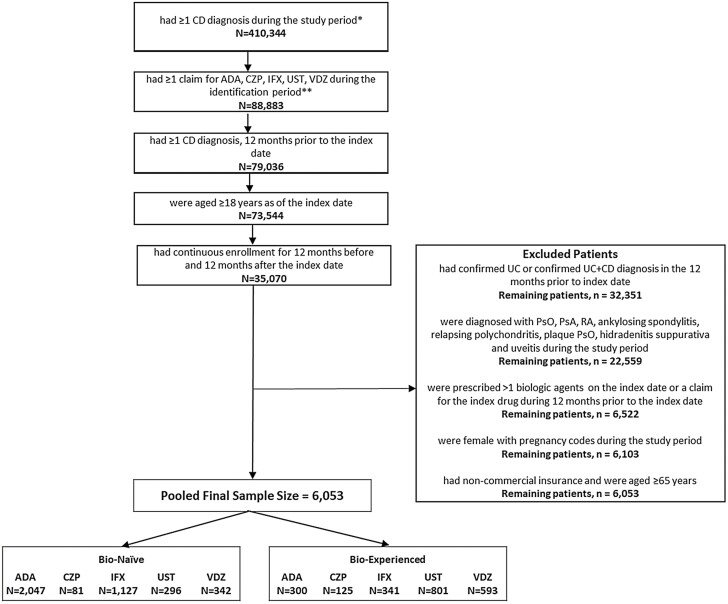
Sample selection. Abbreviations: ADA, adalimumab; CD, Crohn’s disease; CZP, certolizumab; IFX, infliximab; PsA, psoriatic arthritis; PsO, psoriasis; RA, rheumatoid arthritis; UC, ulcerative colitis; UST, ustekinumab; VDZ, vedolizumab. ∗IBM: 26SEP2015-30SEP2018, IQVIA: 26SEP2015-31DEC2018, Optum: 26SEP2015-31MAR2019∗∗IBM: 26SEP2016-30SEP2017, IQVIA: 26SEP2016-31DEC2017, Optum: 26SEP2016-31MAR2018.

Patient selection criteria are described in Figure 1. Patients with ≥1 medical or pharmacy claim for ADA, CZP, IFX, VDZ, or UST from September 26, 2016 to the end of the identification period (Optum: March 31, 2018, IQVIA: December 31, 2017; IBM: September 30, 2017) were identified. The index date was the date of the first medical or pharmacy claim for any biologic treatment during the identification period. Patients were assigned to multiple biologic cohorts based on all the biologics they used during the identification period. Due to small sample size, CZP was excluded from further analysis. Adult patients (≥18 years) with ≥1 CD diagnosis (ICD-9-CM: code 555.xx or ICD-10-CM code K50.xx) during the 12 months prior to the index date were included as long as they had continuous health plan enrollment for 12 months prior to (baseline period) and 12 months after (follow-up period) the index date. Additionally, patients aged ≥65 years on the index date and those with non-commercial insurance were excluded to limit the study population to commercially insured patients where a majority of biologic use occurs. Patients that were diagnosed with psoriasis (PsO), psoriatic arthritis, rheumatoid arthritis, ankylosing spondylitis, relapsing polychondritis, plaque PsO, hidradenitis suppurativa, and uveitis during baseline or follow-up period were excluded from the study. A modified algorithm based on the Manitoba study was used to exclude patients with an ulcerative colitis (UC) diagnosis or those with equal number of UC and CD diagnoses (Supplementary Figure 1).^11,12^

According to the FDA label for UST, moderate-to-severe CD patients should be given a weight-based 6mg/kg intravenous (IV) induction dose followed 8 weeks later by a subcutaneous (SubQ) maintenance dose of 90mg that continues every 8 weeks thereafter. Since the first permanent HCPCS code for the UST IV dose was approved in January 2018, the following algorithm was developed to identify the first IV claim based on unspecified J codes claims (Supplementary Figure 2).^13^ Claims that had an unspecified J code along with CD diagnosis on the same day and a cost range of $1600–$6400 (wholesale acquisition cost of 1 vial was assumed to be $1600^14^; patients would be prescribed anywhere between 2 and 4 vials based on their body weight^15^) were selected during the identification period when no permanent HCPCS codes were available (ie, September 26, 2016–December 31, 2017). Patients were required to have ≥1 identifiable UST claim based on NDC or permanent J code before or after the unspecified J code claim. Additionally, patients were required to have a reasonable service unit that validated that these claims were UST claims. Claims that met the above criteria were classified as UST claims and were included in the analysis.

Pharmacy claims have a variable that identifies days of supply for each claim whereas medical claims do not. Hence, imputation for days of supply was conducted based on the gap between two biological claims. A distribution of the gap between the last of days supplied from the previous claim and the start date of the next claim was evaluated and the following imputation was conducted: if the gap between two claims was <38 days, then days of supply was assumed to be 30 days. If the gap between two claims was between 38 and 46 days, then 42 days was imputed as the days of supply. Finally, if the gap between two claims was >46 days, 56 days (labeled dose) was imputed as the days of supply. No imputation was conducted for ADA since the gap between two claims was generally 30 days with only the quantity dispensed varying.

After applying all the selection criteria, patients were classified into two cohorts:

- *Bio-naive*: Patients that did not have any claim for biologics (ADA, CZP, IFX, natalizumab, UST, or VDZ) during 12 months prior to the index date were classified as bio-naive patients.
- *Bio-experienced*: Patients that had a claim for one of the above drugs during the 12 months prior to the index date were classified as bio-experienced patients.

Among bio-naive and bio-experienced patients, patients were further classified based on the index biologic that they initiated during the identification period.

### Study Measures and Statistical Methods

Patient demographics and clinical characteristics including Charlson comorbidity index (CCI), baseline comorbidities, and concomitant medications were measured during the baseline period. During follow-up, treatment patterns including persistence, discontinuation, medication adherence, and dose titration were evaluated. Persistence was defined as the proportion of patients that remained on the index biologic without a gap of >30 days for ADA, and >120 days for UST, IFX, and VDZ between the run-out date of two biologic claims (gap approximately two times the maintenance dosing interval). Patients who were not persistent to their biologic were classified as discontinuers. Among discontinuers, patients could either switch to another biologic during the follow-up, restart their index biologic, or discontinue without switching or restarting their index biologic. The discontinuation date was defined as the run-out date of the last index medication claim or the switch date, whichever occurred first. To evaluate the robustness of the findings, a fixed discontinuation gap of 90 days was also used to evaluate persistence and discontinuation. Patients typically receive an ADA prescription claim every 4 weeks given its labeled dosing frequency of 2 weeks. Therefore, we evaluated a 60-day gap for ADA as another sensitivity analysis. Additionally, steroid-free persistence—defined as the proportion of patients that either discontinued steroids after their index date or that did not use steroids anytime during the follow-up period (beyond a grace period of 90 days)—was evaluated.

Medication adherence was calculated as medication possession ratio (MPR). MPR was defined as the sum of the days’ supplied of biologic during follow-up divided by the follow-up period. Patients with MPR ≥0.8 were considered as being adherent during the follow-up period. Dose titration (based upon mean maintenance dose) including dose escalation, and dose reduction was assessed among patients with ≥2 maintenance doses in the follow-up period among ADA, UST, and VDZ patients. IFX dose titration was not feasible since IFX dosing is based on patient weight and there is no weight information in the databases. The proportion of patients that had ≥50% dose reduction (ADA: ≤20mg/ 2 weeks; UST: ≤45mg/8 weeks; VDZ: ≤150mg/ 8 weeks), ≥50% dose escalation (ADA: ≥60mg/2 weeks; UST: ≥135mg/8 weeks; VDZ: ≥450mg/8 weeks), and ≥100% dose escalation (ADA: ≥80mg/2 weeks; UST: ≥180mg/8 weeks; VDZ: ≥600mg/8 weeks) from the labeled dose were evaluated. Additionally, a variation of 20% from the labelled dose was used to evaluate dose reduction (ADA: <32mg/2 weeks; UST: <72mg/8 weeks; VDZ: <240mg/8 weeks) and dose escalation (ADA:>48mg/2 weeks; UST: >108mg/8weeks; VDZ: >360mg/8 weeks).

Descriptive analyses were conducted separately for the three data sources and patient populations were pooled to increase the sample size. All analyses were conducted in the pooled population. Means and SDs were computed for continuous variables, and frequency and percentages were calculated for categorical variables.

### Ethical Considerations

This retrospective database analysis did not involve the collection, use, or transmittal of individual identifiable data. As such, Institutional Review Board (IRB) approval to conduct this study was not required and considered exempt according to 45CFR46.101(b)(4): Existing Data & Specimens—No Identifiers. Both the data set itself and the security of the offices where the data are housed meet the requirements of the Health Insurance Portability and Accountability Act (HIPAA) of 1996.

## Results

After applying patient selection criteria, 6053 CD patients were identified across the three databases. Of these, 2047 ADA, 81 CZP, 1127 IFX, 296 UST, and 342 VDZ patients were bio-naive and 300 ADA, 125 CZP, 341 IFX, 801 UST, and 593 VDZ patients were classified as bio-experienced patients (Figure 1). Due to small sample size, CZP patients were excluded from further analysis. The mean age and mean CCI score were consistent across bio-naive and bio-experienced patients with UST (mean age: 39.6–41.0 years; mean CCI: 0.9) and VDZ (mean age: 41.0–41.5 years; mean CCI: 1.0) being older and sicker followed by ADA (mean age: 37.8–38.9 years; mean CCI: 0.7–0.8) and IFX (mean age: 36.5–36.7 years; mean CCI: 0.7; Table 1). Across the cohorts, around 47.4%–56.2% were males (Table 1). Among the comorbidities measured, anemia (ADA: 26.5%–30.7%; IFX: 31.1%–34.0%; UST: 32.1%–41.7%; VDZ: 30.1%–34.1%) and anxiety (ADA: 19.0%–19.1%; IFX: 17.4%–21.1%; UST: 23.0%–25.0%; VDZ: 18.7%–22.1%) were the most prevalent comorbidities during the baseline period. Additionally, among bio-experienced patients, UST patients (16.1%) had a numerically higher proportion of patients with ≥2 biologics during the baseline period followed by VDZ (9.1%), IFX (7.0%), and ADA (7.0%; Table 1).

**Table 1.** Descriptive baseline characteristics in bio-naive and bio-experienced CD patients.

	Adalimumab		Infliximab		Ustekinumab		Vedolizumab	
	Bio-naive	Bio-experienced	Bio-naive	Bio-experienced	Bio-naive	Bio-experienced	Bio-naive	Bio-experienced
Sample size	2047	300	1127	341	296	801	342	593
Age, mean (SD)	38.9 (13.2)	37.8 (13.2)	36.5 (13.4)	36.7 (12.4)	41.0 (12.6)	39.6 (12.8)	41.5 (13.2)	41.0 (12.4)
Age group, *n* (%)								
18–34	827 (40.4%)	125 (41.7%)	548 (48.6%)	168 (49.3%)	95 (32.1%)	299 (37.3%)	113 (33.0%)	185 (31.2%)
35–54	907 (44.3%)	133 (44.3%)	442 (39.2%)	139 (40.8%)	149 (50.3%)	373 (46.6%)	158 (46.2%)	309 (52.1%)
55–64	313 (15.3%)	42 (14.0%)	137 (12.2%)	34 (10.0%)	52 (17.6%)	129 (16.1%)	71 (20.8%)	99 (16.7%)
Gender, *n* (%)								
Male	1123 (54.9%)	162 (54.0%)	633 (56.2%)	175 (51.3%)	155 (52.4%)	422 (52.7%)	178 (52.0%)	281 (47.4%)
Female	924 (45.1%)	138 (46.0%)	494 (43.8%)	166 (48.7%)	141 (47.6%)	379 (47.3%)	164 (48.0%)	312 (52.6%)
Charlson comorbidity index, mean (SD)	0.8 (1.2)	0.7 (1.1)	0.7 (1.1)	0.7 (1.0)	0.9 (1.2)	0.9 (1.2)	1.0 (1.3)	1.0 (1.4)
Comorbidities, *n* (%)								
Anemia	542 (26.5%)	92 (30.7%)	351 (31.1%)	116 (34.0%)	95 (32.1%)	334 (41.7%)	103 (30.1%)	202 (34.1%)
Anxiety	391 (19.1%)	57 (19.0%)	196 (17.4%)	72 (21.1%)	74 (25.0%)	184 (23.0%)	64 (18.7%)	131 (22.1%)
Atherosclerosis	24 (1.2%)	1 (0.3%)	2 (0.2%)	1 (0.3%)	3 (1.0%)	8 (1.0%)	1 (0.3%)	3 (0.5%)
Celiac disease	63 (3.1%)	5 (1.7%)	37 (3.3%)	13 (3.8%)	11 (3.7%)	36 (4.5%)	18 (5.3%)	19 (3.2%)
Cholelithiasis	85 (4.2%)	10 (3.3%)	35 (3.1%)	13 (3.8%)	10 (3.4%)	32 (4.0%)	11 (3.2%)	22 (3.7%)
Chronic pain	119 (5.8%)	13 (4.3%)	53 (4.7%)	21 (6.2%)	26 (8.8%)	57 (7.1%)	16 (4.7%)	36 (6.1%)
Depression	310 (15.1%)	45 (15.0%)	183 (16.2%)	57 (16.7%)	61 (20.6%)	166 (20.7%)	64 (18.7%)	111 (18.7%)
Diabetes	106 (5.2%)	16 (5.3%)	51 (4.5%)	11 (3.2%)	14 (4.7%)	36 (4.5%)	24 (7.0%)	25 (4.2%)
Fatigue	279 (13.6%)	47 (15.7%)	142 (12.6%)	62 (18.2%)	47 (15.9%)	116 (14.5%)	55 (16.1%)	93 (15.7%)
Fistula	118 (5.8%)	22 (7.3%)	117 (10.4%)	42 (12.3%)	24 (8.1%)	110 (13.7%)	19 (5.6%)	43 (7.3%)
Obesity	197 (9.6%)	18 (6.0%)	105 (9.3%)	43 (12.6%)	31 (10.5%)	75 (9.4%)	42 (12.3%)	66 (11.1%)
Venous Thromboembolism	33 (1.6%)	6 (2.0%)	18 (1.6%)	6 (1.8%)	9 (3.0%)	23 (2.9%)	5 (1.5%)	15 (2.5%)
Number of biologics during baseline period, *n* (%)								
Only 1 biologic	—	279 (93.0%)	—	317 (93.0%)	—	672 (83.9%)	—	539 (90.9%)
≥2 biologics	—	21 (7.0%)	—	24 (7.0%)	—	129 (16.1%)	—	54 (9.1%)

**Figure 2.**
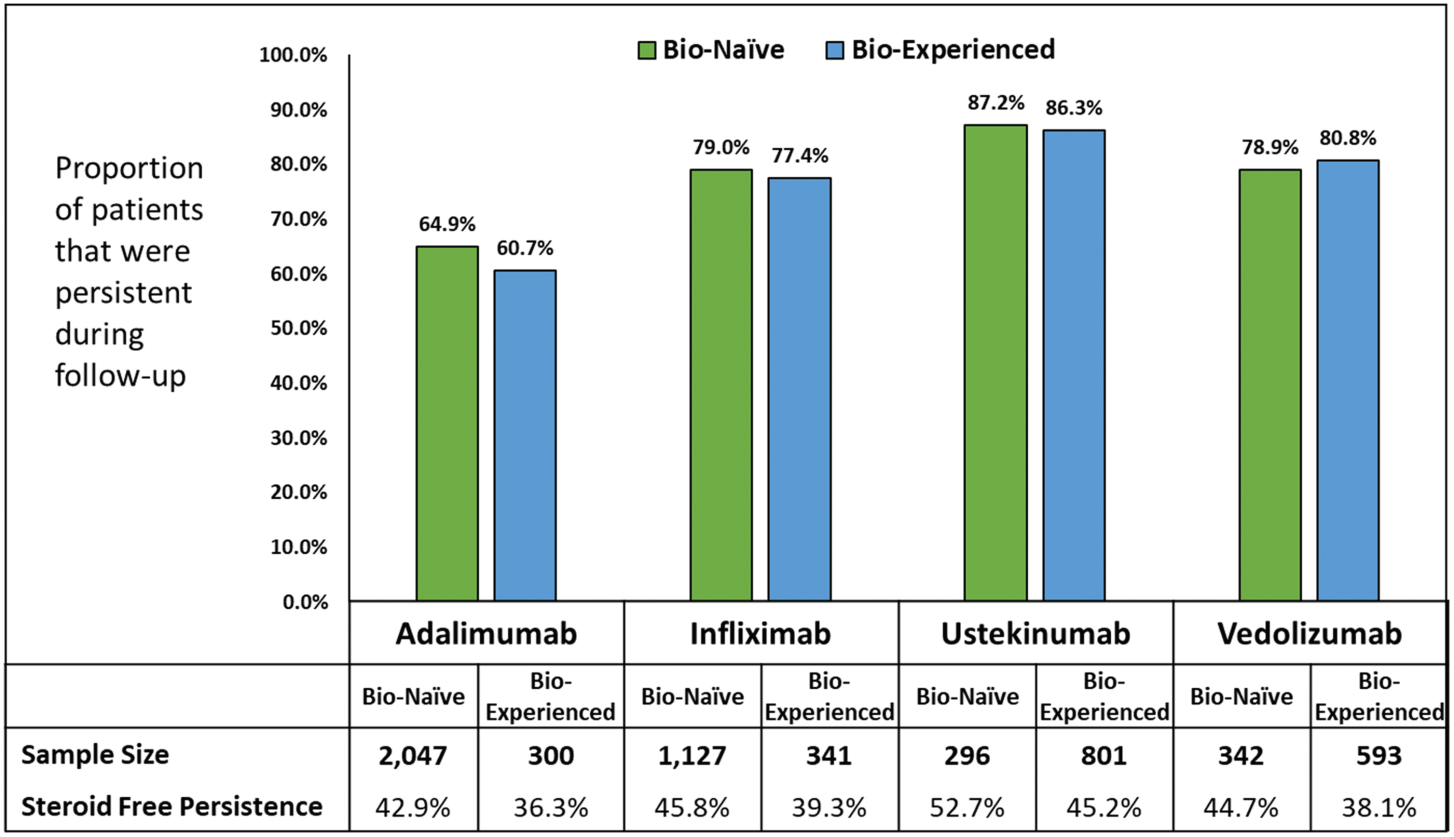
Unadjusted persistence of biologics among bio-naive and bio-experienced CD patients that initiated biologics. Abbreviations: ADA, adalimumab; CD, Crohn’s disease; IFX, infliximab; UST, ustekinumab; VDZ, vedolizumab.

Unadjusted persistence was numerically highest among both bio-naive and bio-experienced UST (87.2%; 86.3%) patients relative to VDZ (78.9; 80.8%), IFX (79.0%; 77.4%), and ADA (64.9%; 60.7%) patients (Figure 2). The trends were similar when sensitivity analyses using a 90 day fixed allowable gap was used across the cohorts although the persistence of ADA increased more than observed with other biologics (Table 2). In addition, the proportion of patients that were persistent and steroid-free were numerically higher among bio-naive patients relative to bio-experienced patients (ADA 42.9% vs 36.3%; IFX 45.8% vs 39.3%; UST 52.7% vs 45.2%; and VDZ 44.7% vs 38.1%), respectively (Figure 2).

**Table 2.** Treatment patterns among bio-naive and bio-experienced CD patients that initiated biologics.

	Adalimumab^c^		Infliximab		Ustekinumab		Vedolizumab	
	Bio-naive	Bio-experienced	Bio-naive	Bio-experienced	Bio-naive	Bio-experienced	Bio-naive	Bio-experienced
Sample size	2047	300	1127	341	296	801	342	593
Main analysis—using a variable gap defintion^a^								
Persistence, *n* (%)	1329 (64.9%)	182 (60.7%)	890 (79.0%)	264 (77.4%)	258 (87.2%)	691 (86.3%)	270 (78.9%)	479 (80.8%)
Discontinuation, *n* (%)	718 (35.1%)	118 (39.3%)	237 (21.0%)	77 (22.6%)	38 (12.8%)	110 (13.7%)	72 (21.1%)	114 (19.2%)
Among patients that discontinued their index biologic, *n* (%)								
Switch, *n* (%)	210 (29.2%)	50 (42.4%)	105 (44.3%)	49 (63.6%)	11 (28.9%)	58 (52.7%)	29 (40.3%)	72 (63.2%)
Adalimumab	0 (0.0%)	0 (0.0%)	49 (20.7%)	5 (6.5%)	3 (7.9%)	13 (11.8%)	6 (8.3%)	15 (13.2%)
Certolizumab pegol	19 (2.6%)	0 (0.0%)	2 (0.8%)	4 (5.2%)	0 (0.0%)	7 (6.4%)	0 (0.0%)	1 (0.9%)
Infliximab	80 (11.1%)	10 (8.5%)	0 (0.0%)	0 (0.0%)	2 (5.3%)	12 (10.9%)	9 (12.5%)	8 (7.0%)
Ustekinumab	58 (8.1%)	28 (23.7%)	23 (9.7%)	31 (40.3%)	0 (0.0%)	0 (0.0%)	14 (19.4%)	48 (42.1%)
Vedolizumab	52 (7.2%)	12 (10.2%)	30 (12.7%)	9 (11.7%)	6 (15.8%)	26 (23.6%)	0 (0.0%)	0 (0.0%)
Restart of index biologic, *n* (%)	282 (39.3%)	40 (33.9%)	0 (0.0%)	0 (0.0%)	0 (0.0%)	1 (0.9%)	0 (0.0%)	0 (0.0%)
Discontinuation without restart or switch, *n* (%)	226 (31.5%)	28 (23.7%)	132 (55.7%)	28 (36.4%)	27 (71.1%)	51 (46.4%)	43 (59.7%)	42 (36.8%)
Sensitivity analysis—using a fixed gap defintion^b^								
Persistence, *n* (%)	1587 (77.5%)	227 (75.7%)	872 (77.4%)	260 (76.2%)	251 (84.8%)	670 (83.6%)	262 (76.6%)	457 (77.1%)
Discontinuation, *n* (%)	460 (22.5%)	73 (24.3%)	255 (22.6%)	81 (23.8%)	45 (15.2%)	131 (16.4%)	80 (23.4%)	136 (22.9%)
Among patients that discontinued their index biologic, *n* (%)								
Switch, *n* (%)	227 (49.3%)	51 (69.9%)	114 (44.7%)	51 (63.0%)	14 (31.1%)	62 (47.3%)	33 (41.3%)	83 (61.0%)
Restart of index biologic, *n* (%)	6 (1.3%)	1 (1.4%)	0 (0.0%)	1 (1.2%)	2 (4.4%)	8 (6.1%)	1 (1.3%)	2 (1.5%)
Discontinuation without restart or switch, *n* (%)	227 (49.3%)	21 (28.8%)	141 (55.3%)	29 (35.8%)	29 (64.4%)	61 (46.6%)	46 (57.5%)	51 (37.5%)
Medication Possession Ratio, mean (SD)	0.8 (0.3)	0.8 (0.3)	0.9 (0.4)	0.9 (0.4)	0.9 (0.3)	0.9 (0.3)	0.9 (0.4)	0.9 (0.3)
Patients with MPR ≥ 0.8, *n* (%)	1350 (66.0%)	196 (65.3%)	801 (71.1%)	247 (72.4%)	204 (68.9%)	568 (70.9%)	233 (68.1%)	430 (72.5%)

A variable discontinuation gap of >30 days for ADA and >120 days for UST, IFX, and VDZ between the run-out date of two biologic claims (gap approximately two times the maintenance dosing interval) was used to define discontinuation.

A fixed gap of 90 days between the run-out date of two biologic claims was used to define discontinuation as sensitivity analysis.

Another sensitivity analysis using a 60-day gap definition was evaluated for ADA. Among bio-naive patients, 67.1% were persistent on ADA and 32.9% discontinued ADA during the follow-up (Among patients that discontinued, 33.9% switched biologics, 27.8% of patients discontinued then restarted ADA, and 38.3% discontinued without restart or switch); whereas for bio-experienced ADA patients persistence was observed in 65.7%, and 34.3% patients discontinued ADA during 12 months, (Among patients that discontinued, 50.5% switched biologics, 24.3% of patients discontinued then restarted ADA, and 25.2% discontinued without restart or switch).

Abbreviations: MPR: Medication Possession Ratio.

Among bio-experienced patients that discontinued their index biologic, many patients switched treatment (ADA: 42.4%; IFX: 63.3%; UST: 52.7%; VDZ: 63.2%), with UST (23.7-42.1%) being the most common drug patients switched to (Table 2). Among bio-naive patients that discontinued their index biologic, many discontinued without restart or switch to another biologic during the follow-up period (ADA: 31.5%; IFX: 55.7%; UST: 71.1%; VDZ: 59.7%; Table 2). The proportion of ADA patients restarting their index biologic after discontinuation (39.3% in bio-naive and 33.9% in bio-experienced patients)—was numerically higher than that seen for IFX, UST, and VDZ in the base case which used a gap of 30 days for ADA and 120 days for IFX, UST, and VDZ. To check the robustness of these findings, a gap of 60 days was used to evaluate persistence across ADA patients. The trends were generally consistent although the proportion of patients that were persistent increased slightly, with results for bio-naive patients similar to those observed in bio-experienced patients (bio-naive: 67.1% persistent on ADA and 32.9% discontinued ADA during the follow-up [Among patients that discontinued, 33.9% switched biologics, 27.8% of patients discontinued then restarted ADA, and 38.3% discontinued without restart or switch]; bio-experienced patients: 65.7% persistent on ADA and 34.3% discontinued ADA during 12 months, [Among patients that discontinued, 50.5% switched biologics, 24.3% of patients discontinued then restarted ADA, and 25.2% discontinued without restart or switch]).

During the follow-up period, mean MPR was similar across bio-naive and bio-experienced patients as well as across the biologic cohorts (mean MPR 0.8–0.9; Table 2). Bio-naive patients had a numerically lower proportion of adherent patients across IFX (71.1% vs 72.4%) UST (68.9% vs 70.9%), VDZ (68.1% vs 72.5%) and ADA (66.0% vs 65.3%) than bio-experienced patients (Table 2).

Among patients with ≥2 maintenance doses, 50% dose reduction was observed in 1.1%–1.2% of ADA, 1.4%–1.5% of UST, and 0.0%–0.2% of VDZ patients whereas 50% dose escalation was observed in 11.1%–12.0% of ADA, 8.3%–8.8% of UST, and 3.5%–6.4% of VDZ patients across bio-naive and bio-experienced patients (Table 3). When 20% variation was used, the proportion of bio-experienced patients that had dose escalation was numerically higher than observed in bio-naive patients for UST (16.9% vs 13.4%, respectively) and VDZ patients (14.7% vs 12.4%, respectively), while it remained similar for bio-naive and bio-experienced ADA patients (16.1% vs 16.4%, respectively).

**Table 3.** Unadjusted dose titration among bio-naive and bio-experienced Crohn’s disease patients that initiated biologics.

Adalimumab	Doses	Bio-naive *N* = 1790	Bio-experienced *N* = 267
50% Dose escalation from labelled maintenance dose	≥60mg/2 weeks	199 (11.1%)	32 (12.0%)
100% Dose escalation from labelled maintenance dose	≥80mg/2 weeks	44 (2.5%)	4 (1.5%)
50% Dose reduction from labelled maintenance dose	≤20mg/ 2 weeks	22 (1.2%)	3 (1.1%)
Using 20% variation—patients on label	[32–48] mg/2 weeks	1356 (75.8%)	201 (75.3%)
Using 20% variation—patients with dose escalation	>48mg/2 weeks	294 (16.4%)	43 (16.1%)
Using 20% variation—patients with dose reduction	<32mg/2 weeks	140 (7.8%)	23 (8.6%)
Ustekinumab	Doses	Bio-naive *N* = 276	Bio-experienced *N* = 744
50% Dose escalation from labelled maintenance dose	≥135mg/8 weeks	23 (8.3%)	69 (8.8%)
100% Dose escalation from labelled maintenance dose	≥180mg/8 weeks	9 (3.3%)	17 (2.2%)
50% Dose reduction from labelled maintenance dose	≤45mg/8 weeks	4 (1.4%)	12 (1.5%)
Using 20% variation—patients on label	[72–108] mg/8weeks	214 (77.5%)	597 (76.2%)
Using 20% variation—patients with dose escalation	>108mg/8weeks	37 (13.4%)	132 (16.9%)
Using 20% variation—patients with dose reduction	<72mg/8 weeks	25 (9.1%)	54 (6.9%)
Vedolizumab	Doses	Bio-naive *N* = 226	Bio-experienced *N* = 422
50% Dose escalation from labelled maintenance dose	≥450mg/8 weeks	8 (3.5%)	27 (6.4%)
100% Dose escalation from labelled maintenance dose	≥600mg/8 weeks	1 (0.4%)	2 (0.5%)
50% Dose reduction from labelled maintenance dose	≤150mg/ 8 weeks	0 (0.0%)	1 (0.2%)
Using 20% variation—patients on label	[240–360] mg/8 weeks	187 (82.7%)	342 (81.0%)
Using 20% variation—patients with dose escalation	>360mg/8 weeks	28 (12.4%)	62 (14.7%)
Using 20% variation—patients with dose reduction	<240mg/8 weeks	11 (4.9%)	18 (4.3%)

## Discussion

To the authors’ knowledge, this is the first study that descriptively analyzed the treatment patterns for most of the biologics approved for CD during the study period. This retrospective observational study integrated information from three large US commercial databases to provide a descriptive assessment of real-world biologic persistence, adherence, and dose titration among CD patients. During the follow-up, unadjusted persistence rates were numerically higher for UST followed by VDZ, IFX, and ADA. Among patients that discontinued their index biologic most bio-naive patients discontinued without restart or switch whereas most bio-experienced patients switched to another biologic when a variable gap definition was used. These trends were consistent across bio-naive and bio-experienced patients when a fixed gap definition was used as well. Additionally, the proportion of patients that were persistent and steroid-free was also numerically higher in bio-naive compared with bio-experienced patients, while dose escalation was lower across most cohorts.

Persistence to biologic treatment and reduction in switching to another biologic is generally used as a proxy for efficacy and safety/tolerability which relates to the risk-benefit profile of the treatment.^7,16,17^ A systematic literature review conducted using electronic databases for 2012–2017 reported that 38%–77% of IBD patients were found to be non-persistent (discontinued treatment) to anti-TNF biologics.^16^ During the 12-month follow-up period, 7%–65% of patients discontinued their index biologic and 4.5%–20% switched to another biologic.^16^ A real-world study among TNF-inhibitors (ADA, CZP, IFX) reported that 38%–45% of CD patients discontinued/switched their anti-TNF therapy within 12 months of treatment initiation.^18^ The current study reported slightly lower proportion of discontinued/switched patients—35.1%–39.3% of ADA patients and 21.0%–22.6% of IFX using a variable gap definition. These differences could be attributed to the different gap definitions used to identify discontinuation/switch—Rubin et al used a gap of ≥30 days whereas the current study used a variable gap definition (>30 days for ADA and >120 days for IFX) as well as a fixed gap definition of 90 days.

Suboptimal treatment, including loss of response, in chronic illness, has been associated with poor humanistic and economic outcomes.^19^ In the current study, 50% dose escalation rates were observed in 3.5%–12.0% while 50% dose reduction rates were 0%–1.5% with UST patients having a numerically higher dose reduction rate. This could be attributed to a higher proportion of UST patients (~16%) that used 2 or more biologics during the baseline period. A real-world study reported a 50% increase in hospitalization rate and steroid use in IBD patients with poor adherence to SubQ biologics.^20^ Another real-world study examining UST treated CD patients reported dose-escalation among 17.9% and dose reduction among 5.1% CD patients.^11^ Other studies among CD patients using claims databases reported a dose escalation rate of 17%–24%.^7,21^ Heterogeneity between the dose titration rates could be attributed to definitions of treatment failure, study sample sizes, study designs, and follow-up periods across the studies.

The approval of new biologics continues to change the treatment landscape for CD. This study adds to existing literature by describing demographic and clinical characteristics for patients treated with each biologic, as well as treatment patterns and persistence associated with each biologic. Additionally, this study aids in addressing some of the data gaps regarding the use of UST and other biologics in bio-naive patients by pooling patients from three national claims databases to obtain larger cohorts. A better understanding of patient characteristics and treatment patterns associated with existing biologics may be informative to clinicians managing CD patients, and also to payers and those who make decisions regarding access and utilization management of biologics. Future studies comparing the treatment effectiveness and adverse event rates across different biologics will further assist stakeholders in making informed decisions regarding treatment strategies for CD patients.

As with all retrospective, observational analyses, this study was limited to determining associations and causality cannot be inferred. As this study was descriptive in nature, results should be interpreted with caution when comparing biologics, as no adjustments for confounding were made. In addition, administrative claims data are not clinically comprehensive and may contain coding errors and missing information. NDC and HCPCS codes were used to identify UST claims. Permanent HCPCS codes for IV UST were not available for most of the identification period; hence, an algorithm (using cost per UST claim with reasonable service units provided) was used to identify unspecified J codes that could be used as a proxy for UST IV induction dose. This algorithm was based on an approach by Hudesman et al. using a UC or CD diagnosis and costs to identify unspecified claims for vedolizumab.^13^ This could have an influence on the persistence rates because of reduced accuracy in identifying the first IV induction UST claim. Future validation of this algorithm is warranted. Additionally, ICD codes based on inpatient and outpatient claims were used to identify CD patients rather than laboratory confirmatory tests. It is possible that some of the patients were misclassified as CD patients. Retrospective claims databases are designed for payment and reimbursement purposes and have certain inherent limitations for research. Insurance medical claims does not have indication on the number of days the medication was dispensed for. Additionally, the dispensed date for pharmacy claims do not necessarily correspond to the date the medication was administered to the patient. Therefore, data imputation was conducted to identify the days of supply of each infusion claim. This may result in over or under-representation of persistence and dose titration rate especially for infusion biologics such as IFX and VDZ. The reasons for drug discontinuation, or switch to another medication are not documented in claims databases. Hence, factors influencing treatment patterns were unknown. Bio-naive designation is based on the presence of a biologic claim in the 12-month baseline period. It is possible that patients identified as bio-naive in this analysis may have had biologic use prior to the 12-month baseline period. We also followed patients for a fixed 12 months from their index date, so it is possible that patients discontinuing their index biologic closer to the end of the 12-month follow-up may have switched to another biologic after the end of our observation period. Thus, the proportion of discontinuers switching to another biologic may be under-reported. Further, it is possible that delayed doses or use of drug samples by the physician may appear as dose reductions in our analysis. Use of drug samples are generally not captured in the claims data sources used in our study and their use may lead to longer periods before subsequent claims are observed, thus appearing as dose reductions for such patients. Therefore, dose titration results should be interpreted with these limitations in mind. This study did not incorporate complete public insurance databases (ie, Medicare or Medicaid); hence, the findings may not be generalizable to the entire population of the United States.

## Conclusions

This large, retrospective, real-world study using pooled claims data for commercially insured patients with CD examined persistence, discontinuation, and dose titration of biologic therapies and found that descriptive persistence rates were numerically highest for UST, followed by VDZ, IFX, and ADA. The results were generally similar across different discontinuation gap definitions and across bio-naive and bio-experienced patients. Dose escalation was numerically highest for ADA followed by UST and VDZ. Generally, the proportion of patients that had dose escalation was numerically higher for bio-experienced relative to bio-naive patients. Given the limitations of descriptive claims database analysis, further studies that adjust for confounders are needed to examine biologic treatment patterns, and longer periods of observation should also be considered.

## Supplementary Material

otab076_suppl_Supplementary_Materials_1

## Data Availability

Data not publicly available.
