# Supplementary material for: Persistence, Dosing, and Other Treatment Patterns Among Crohn’s Disease Patients Initiating Biologics in United States
Source: Crohns Colitis 360. 2021 Nov 5;3(4):otab076. doi: 10.1093/crocol/otab076 (PMC9802353; doi:10.1093/crocol/otab076)
Supplement: otab076_suppl_Supplementary_Materials_1 [file otab076_suppl_supplementary_materials_1.docx]

**Supplemental Data**

**Supplemental Figure 1. Identifying Confirmed CD Patients**

**
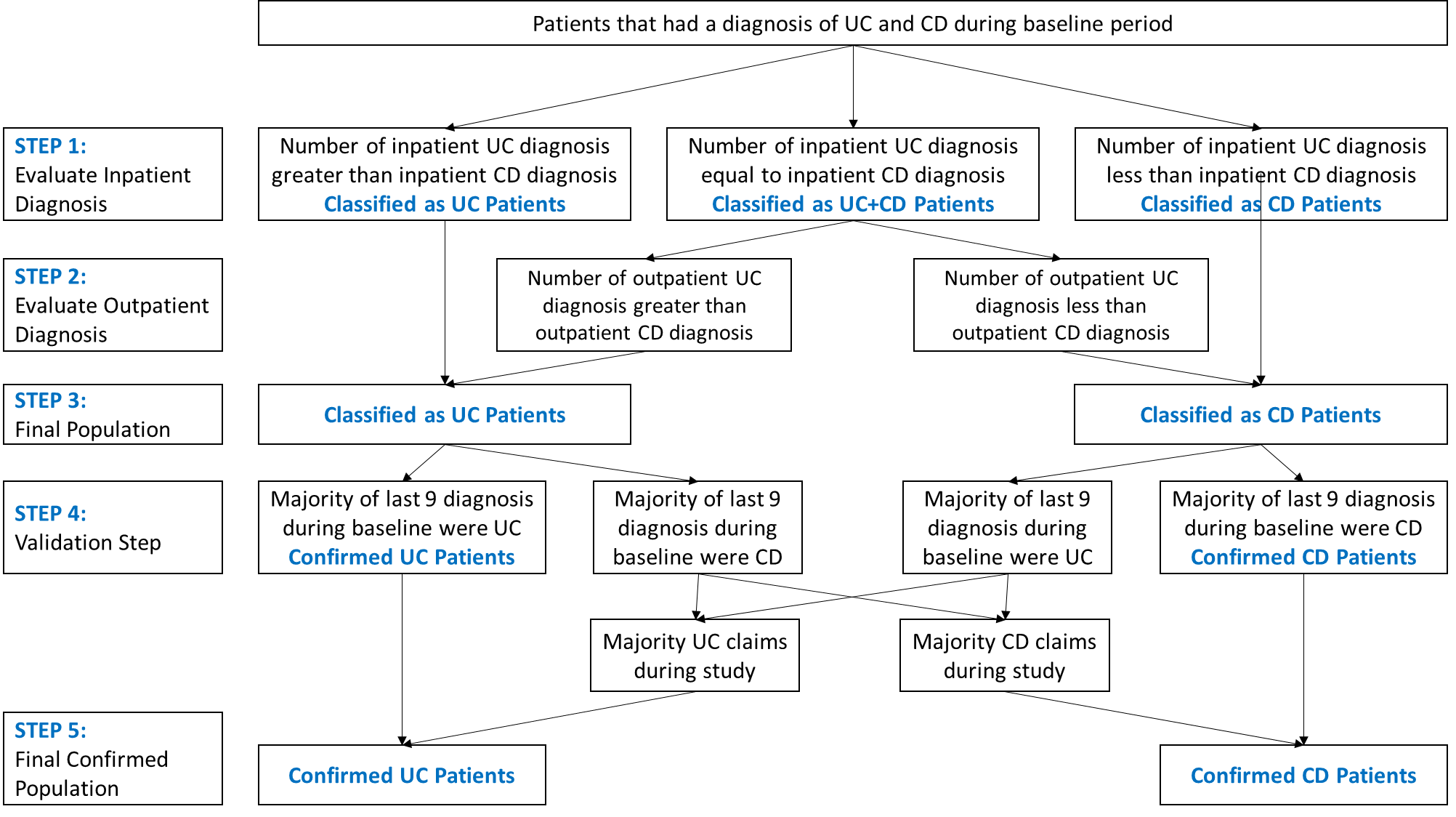
**

CD: Crohn's disease; UC: Ulcerative Colitis

**Supplemental Figure 2. Identifying Unspecified UST Claims**


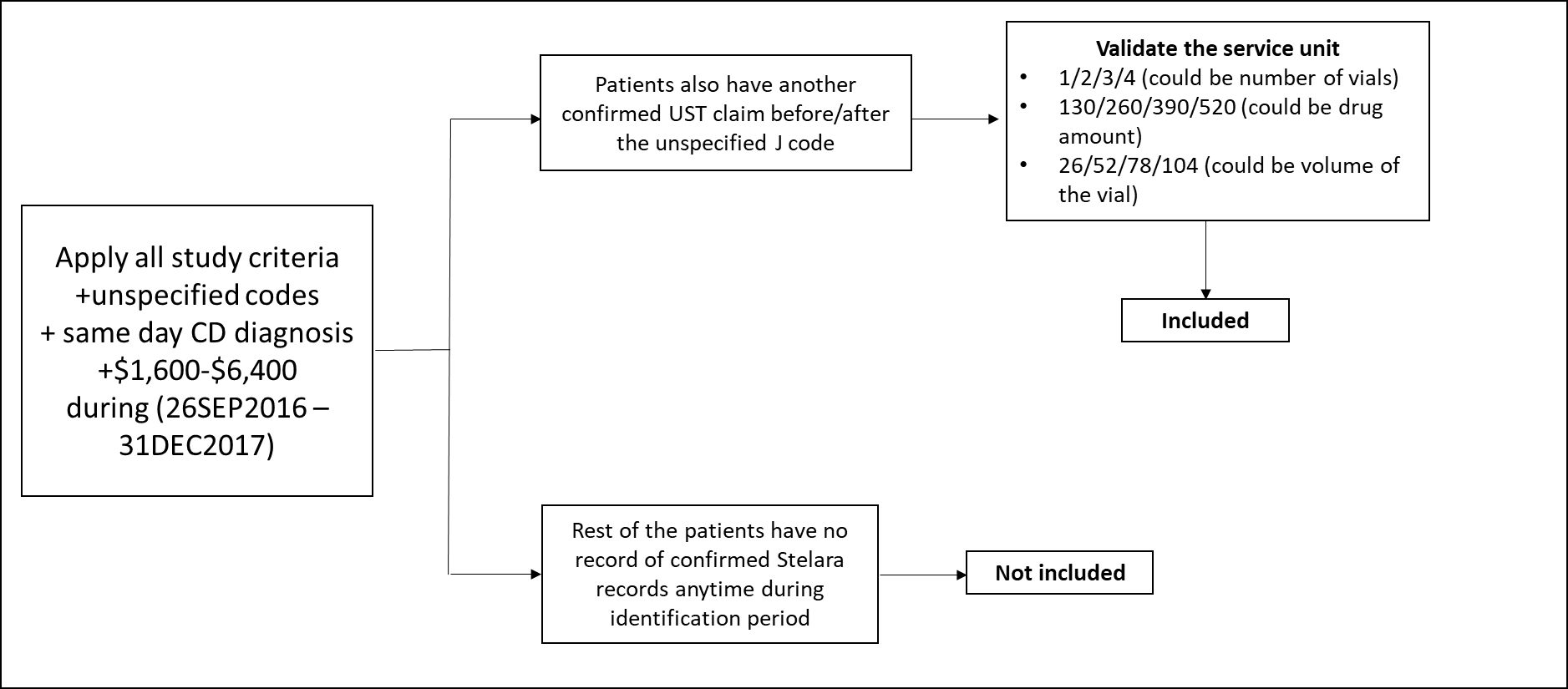


CD: Crohn's disease; UST: UST: ustekinumab
